# Supplementary material for: Learning curve and surgical outcome of robotic assisted colorectal surgery with ERAS program
Source: Sci Rep. 2022 Nov 29;12:20566. doi: 10.1038/s41598-022-24665-w (PMC9709162; doi:10.1038/s41598-022-24665-w)
Supplement: Supplementary file 2 — Supplementary Information 2. [file 41598_2022_24665_MOESM2_ESM.zip › RAL Raw data-Ñ[▒K/121/OP N_ñw╝╨░O▒Kñσ.pdf]

W52 / W52

臺中榮民總醫院
手術室護理記錄

[redacted]

基本資料

- 室別： 開刀房-17室，手術科別： CRS，手術類別： 常規，日期： 2021/10/28
- 進入時間： 1330 ； 麻醉開始時間： 1355 ； Time Out時間： 1440 ； 劃刀時間： 1440 ； 手術結束時間： 1830 送出時間： 1855 ；
- 麻醉方式： GE
  診斷： Malignant tumor of rectum
  手術名稱： Robot Assisted Colectomy(L)
  手術局部用藥： NIL
  術前預防性抗生素：
  - 抗生素名稱一： Veterin 1GM IVD/由麻醉護理師給予
  - 時間： 1435
  - 上次簽章: 陳盈妤
  ，追加預防性抗生素：
- 手術醫師： 蔣鋒帆，陳瑨夫，陳凱祥
- 麻醉醫師： VS 齊光彪
-
  - 刷手護士A ： 陳妍希，時間： 1330，刷手護士B ： 黃彥翔，時間： 1330，接替刷手一： 陳心慈/林愛甯，時間： 1500，接替刷手二： 陳心慈，時間： 1700
  - 流動護士A ： 陳盈妤，時間： 1330，接替流動一： 許芷瑄，時間： 1500，接替流動二： 林怡廷，時間： 1900
- 更多接替及流動護士： 接替刷手三： 林愛甯，時間： 1730

皮膚狀況

A-V Shunt： 無
術前： 完整
記錄者： 上次簽章: 陳盈妤
術後： 完整
記錄者： 林怡廷

手術準備

手術臥式 單· ·手術臥式

-
  - 手術臥式： 仰臥，NIL
  - 使用附件： 溫毯 40 ℃，其它： 海綿側身用物
  - 皮膚準備： 10%Aq Betadine，Hibiscrub，2% Chlorhexidine，其它： NIL
  - 電燒： 使用電燒: 電極片位置： 其它： 導電軟墊，其它： NIL，記錄者： 上次簽章: 陳盈妤

止血帶T： 無

植入物

無

標本送檢

- 細菌培養 無
- 細胞學 無
- 冰凍切片 無
- 病理標本
  組織名稱一： S-colon
  ,
  組織名稱二： IMA root
  ,
  組織名稱三： Distal cut end
  ，更多組織名稱
  -

- ○ 組織名稱四： peritoneum
- 截肢 無

標本交付 標本： 4 袋 ， 標本： 0 盒 ， 標本： 0 瓶 ， 細胞學： 0 管 ， Culture Tube： 0 管 ， 被交付醫師： CR 陳璿夫 ，

- 記錄者： 上次簽章: 許芷瑄
- 時間： 1825

傷口術後敷料

傷口敷料：　　　　傷口敷料： 無

- 請於圖上標示部位：

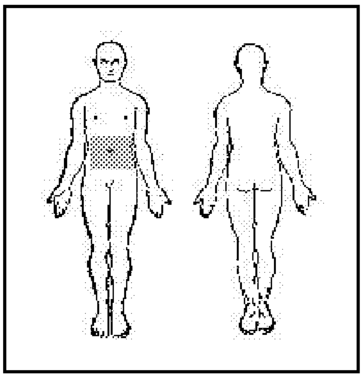

- 傷口敷料： Trocar:menport

導管

導管	天數	導管資訊	插入日期	插入時間	更換日期	拔除日期	拔除時間	拔除原因	預計拔除/更換	預計拔除/更換日期
Foley(1)	1	號碼:16 Fr.; 部位:膀胱;	2021/10/28	1420						
Epidural PCA(1)	1	部位:硬脊外;	2021/10/28	1400						
Double Lumen CVC(1)	1	號碼:8Fr. ; 部位:; 象限:RU; 註記:fix 15cm;	2021/10/28	1415						
A-line (1)	1	號碼:20 ; 部位:左手;	2021/10/28	1415						

用物計數

- 手術中出血量： 100 c.c.
- 手術中尿量： 550 c.c.
-
  - ○ 其它： 無
  - ○ NIL

用物計數： 多切口同時縫合

- 1.器械： 正確 ，2.縫針： 正確 ，3.刀片： 正確 ，4.紗布： 正確 ，5.腹部墊： 未使用 ，6.紗球： 未使用 ，7.PEANUTS ： 未使用 ，8.紗條： 未使用 ，9.絲帶： 未使用 ，10.針頭： 未使用 ，11.Vessel loop： 未使用 ，12.Boots： 未使用 ，13.Jelco： 未使用 ，14.棉條： 未使用 ，
  - ○ 第一次：
    - ■ 刷手護士 ： 林愛甯
    - ■ 流動護士： 上次簽章: 許芷瑄
    - ■ 時間： 1800

  ,
  - ○ 第二次：
    - ■ 刷手護士 ： 林愛甯
    - ■ 流動護士： 上次簽章: 許芷瑄
    - ■ 時間： 1805

  ,
  - ○ 第三次：
    - ■ 刷手護士 ： 林愛甯
    - ■ 流動護士： 上次簽章: 許芷瑄

- 時間： 1810

備註

備註： Davinci XI 30度:NO.1

- 記錄者： 上次簽章: 陳盈妤
- 時間： 1440

填寫護理人員 林怡廷

正式摘要

W52 / W52

doctorRank:B
